# Mental- and physical-health comorbidity are strongly associated in idiopathic inflammatory myopathies: a multinational cross-sectional e-survey

**DOI:** 10.1007/s00296-026-06262-6

**Published:** 2026-08-29

**Authors:** Samuel Katsuyuki Shinjo, Lekshmi Minikumari Rahulan, Meera Shah, Anchit Chauhan, Suhana Hussain, Paula Jordan, Meghna Lama, Oladipo Kunle Afolayan, Vincenzo Venerito, Tamar B. Rubinstein, Ioannis Parodis, Elena Nikiphorou, Joanna Makowska, Aleksandra Opinc-Rosiak, Rada Miskovic, Marcin Milchert, Dimitri Luz Felipe da Silva, COVAD Study Group, Odirlei Andre Monticielo, Simone Appenzeller, Vidya Sadanand Limaye, Binit Vaidya, Ai Lyn Tan, Nelly Ziade, Tsvetelina Velikova, Abraham Edgar Gracia-Ramos, Masataka Kuwana, Johannes Knitza, Ashima Makol, Carlos Enrique Toro Gutiérrez, Carlo Vinicio Caballero-Uribe, Dzifa Dey, Jessica Day, Laura Andreoli, Chris Wincup, Nicola Dalbeth, Gerd-Rüdiger Burmester, Guochun Wang, Lorenzo Cavagna, Vahed Maroufy, Vikas Agarwal, Latika Gupta

**Affiliations:** 1 Division of Rheumatology, Faculdade de Medicina FMUSP, Universidade de São Paulo, São Paulo, SP Brazil; 2 Department of Clinical Immunology and Rheumatology, Sanjay Gandhi Postgraduate Institute of Medical Sciences, Lucknow, India; 3 Department of Rheumatology, Topiwala National Medical College and B.Y.L Nair Hospital, Mumbai, India; 4 Maulana Azad Medical College, New Delhi, India; 5 Mahatma Gandhi Medical College and Hospital, Jaipur, India; 6 Honorary General Secretary, Myositis, UK; 7 The University of Texas Health Science Center at Houston School of Public Health: Houston, Texas, USA; 8 Rheumatology Unit, Department of Precision and Regenerative Medicine-Ionian Area, University of Bari “Aldo Moro”, Bari, Italy; 9 Division of Rheumatology, Albert Einstein College of Medicine, 1300 Morris Park Avenue, Bronx, USA; 10 Division of Rheumatology, Department of Medicine Solna, Center for Molecular Medicine (CMM), Karolinska Institutet, Karolinska University Hospital, Stockholm, Sweden; 11 Department of Rheumatology, Faculty of Medicine and Health, Örebro University, Örebro, Sweden; 12 Centre for Rheumatic Diseases, King’s College London, London, UK; 13 Rheumatology Department, King’s College Hospital, London, UK; 14 Department of Rheumatology, Medical University of Lodz, Zeromskiego 113, 90-549 Lodz, Poland; 15 Clinic of Allergy and Immunology, Faculty of Medicine, University Clinical Centre of Serbia, University of Belgrade, Belgrade, Serbia; 16 Department of Internal Medicine, Rheumatology, Diabetology, Geriatrics and Clinical Immunology of Pomeranian Medical University in Szczecin, Szczecinie, Poland; 17 University Santo Amaro, Hospital Israelita Albert Einstein, São Paulo, Brazil; 18 Department of Biostatistics and Data Science, School of Public Health, The University of Texas Health Center at Houston, Austin, TX 77002 USA; 19 Department of Rheumatology, Royal Wolverhampton Hospitals NHS Trust, Wolverhampton, UK; 20 Department of Inflammation and Ageing, School of Infection, Inflammation and Immunology, University of Birmingham, Edgbaston, B15 2TT UK; 21 Francis Crick Institute, London, UK

**Keywords:** Multimorbidity, Myositis, Inclusion body, Patient reported outcome measures, Cluster analysis, Morbidity, Logistic models

## Abstract

Comorbidities are associated with morbidity, function, and survival in idiopathic inflammatory myopathies (IIM), but subtype-specific patterns, mental-physical comorbidity relationships, and patient-reported outcomes remain incompletely characterized. We hypothesized that multimorbidity in IIM is heterogeneous, associated with demographic, clinical, and psychosocial factors, and linked to differences in physical function and fatigue. We studied 1028 adults with self-reported, specialist-confirmed IIM. Comorbidities were assessed using the Functional Comorbidity Index and categorized into basic, complex, mental-health, and autoimmune domains. Logistic regression identified factors associated with mental- and physical-health comorbidity; clustering defined comorbidity patterns. Participants were predominantly female (71.3%), median age 59.0 years. Overall, 46.7% had basic multimorbidity, 15.4% complex multimorbidity, 33.6% mental-health comorbidity, and 14.7% autoimmune multimorbidity. Interstitial lung disease was concentrated in anti-synthetase syndrome (50.7%) and polymyositis (42.6%), while hypertension (37.8%) and malignancy (35.0%) were concentrated in inclusion body myositis. Chronic pain/fatigue syndromes were associated with mental-health burden (63.2% vs. 27.5%, p < 0.001). Mental-health comorbidity was associated with younger age (OR 0.98/year), complex multimorbidity (OR 2.85), immunosuppressive therapy (OR 1.53), and lower family support (OR 0.94/unit). Physical-health comorbidity was independently associated with older age (OR 1.05/year), mental-health comorbidity (OR 3.96), and overlap myositis (OR 3.33). Clustering identified three multimorbidity profiles: low-burden (49.3%), cardiometabolic/malignancy-predominant with low mental-health burden (22.9%), and pain-fatigue/mental-health-predominant (27.7%), which differed significantly in physical function and fatigue. Multimorbidity in IIM is common, heterogeneous, and shows a strong concurrent relationship between mental- and physical-health comorbidity, supporting integrated mental-health screening and subtype-tailored surveillance.

**Supplementary Information:**

The online version contains supplementary material available at https://doi.org/10.1007/s00296-026-06262-6.

## Introduction

Idiopathic inflammatory myopathies (IIM) are rare systemic autoimmune diseases characterized by chronic muscle inflammation and diverse extra-muscular manifestations [1, 2]. Distinct clinical and serological subsets include dermatomyositis (DM), juvenile DM (JDM), polymyositis (PM), inclusion body myositis (IBM), anti-synthetase syndrome (ASyS), immune-mediated necrotizing myopathy (IMNM), cancer-associated myositis, and overlap myositis (OM) [1, 2]. Beyond muscle disease, patients frequently develop comorbidities that contribute substantially to disability and disease burden.

Comorbidities are strongly associated with morbidity, quality of life, and survival in IIM [3–7]. Their development reflects interactions between disease-related inflammation, treatment exposures, particularly glucocorticoids [8], and psychosocial factors. Mental health disorders, including anxiety and depression are associated with greater disease burden and may affect physical comorbidity [9, 10].

Although multimorbidity has been described in IIM [10], important gaps remain. Previous studies have not comprehensively evaluated the relationship between mental-health and physical-health comorbidity, the contribution of pain, fatigue, lifestyle, and psychosocial factors, or patterns of comorbidity clustering. Understanding these relationships may improve risk stratification and patient care. We hypothesized that multimorbidity is heterogeneous, that mental-health comorbidity is independently associated with physical-health comorbidity, and that distinct multimorbidity profiles are associated with differences in physical function and fatigue. Accordingly, we aimed to characterize comorbidity patterns in IIM, identify factors associated with mental- and physical-health comorbidity, and define multimorbidity profiles via clustering.

## Materials and methods

### Study design and data source

This cross-sectional study used data from a multinational, validated, self-administered e-survey (Collating the Voice of Autoimmune Diseases Study) designed to capture well-being, comorbidity burden, and associations of health among individuals with systemic autoimmune diseases [11]. The survey was administered electronically via SurveyMonkey (SurveyMonkey Inc., San Mateo, CA, USA). It was disseminated broadly through patient organizations and clinical networks rather than to a fixed, enumerable sampling frame; a conventional response rate could not be calculated. Reporting followed established recommendations for designing, conducting, and reporting survey research [12]. Its development, content, and methodology have been described in detail elsewhere [11].

### Study population

Adults (≥ 18 years) who completed the survey and provided informed consent were eligible. Only participants reporting a self-reported, specialist-confirmed diagnosis of IIM were included; diagnoses based solely on self-report or confirmation by a general practitioner were excluded to strengthen diagnostic validity. Classification criteria, autoantibody data, and medical record verification were unavailable. Eligible IIM subtypes comprised DM, JDM, PM, IBM, IMNM, ASyS, and OM. Participants with missing data on age, sex, IIM subtype, disease activity status, or any Functional Comorbidity Index (FCI) item required to operationalize the multimorbidity constructs were excluded (Online resource 1). For patient-reported outcome measures and lifestyle variables, we performed a complete case analysis within each model (e.g., listwise deletion restricted to the variables used in that analysis), without imputation, as the proportion of missingness per variable was low (< 10% for all variables included in multivariable models).

### Variables and definitions

Comorbidities were measured using the FCI [13], a validated, self-reported 18-item instrument originally developed and tested in community-based adult populations to predict physical function as the primary outcome, in contrast to other comorbidity indices (e.g., the Charlson Comorbidity Index) that were designed to predict mortality. The FCI includes the following directly reported chronic conditions: arthritis, osteoporosis, asthma, chronic obstructive pulmonary disease, angina, congestive heart failure, myocardial infarction, other cardiovascular disease (including peripheral vascular disease), stroke, diabetes mellitus, obesity, gastrointestinal disease (including peptic ulcer disease), liver disease, kidney disease, visual impairment, hearing impairment, and depression/anxiety/emotional problems [13]. These conditions were captured item-by-item to compute the FCI score and construct organ-system-specific comorbidity domains.

The FCI has demonstrated acceptable reliability when collected via patient questionnaire, with high intra-rater reliability (ICC = 0.90) and moderate inter-rater reliability, and has shown criterion validity for predicting long-term physical function and health-related quality of life across diverse clinical populations [13]. Its focus on functional outcomes and suitability for self-administration make it well suited to patient-reported survey studies such as this one. Because the FCI was developed for general medical populations, it does not include items specific to myositis-associated organ involvement. To address this, the survey supplemented the core FCI items with myositis-relevant conditions including interstitial lung disease (ILD) and malignancy, captured as discrete self-reported items and incorporated into the respiratory and neoplastic comorbidity domains, respectively, alongside the FCI-derived domains. Cardiac involvement was natively captured within the FCI and required no supplementation. Treatment-related complications (e.g., glucocorticoid-induced diabetes) could not be distinguished from disease-related comorbidity.

Multimorbidity was operationalized using four complementary definitions: (i) WHO multimorbidity, defined as ≥ 2 co-existing chronic conditions [14]; (ii) basic multimorbidity, defined as ≥ 2 non-rheumatic chronic conditions [15]; (iii) complex multimorbidity, defined as ≥ 3 chronic conditions affecting ≥ 3 distinct organ systems [15]; and (iv) autoimmune multimorbidity, defined as co-existence of ≥ 3 systemic autoimmune diseases [15]. For the purpose of our analyses, “organ systems” for complex multimorbidity were defined a priori as respiratory, renal, hepatobiliary, gastrointestinal, reproductive, special sensory, musculoskeletal, cardiovascular, central nervous system, endocrinological, neoplastic, chronic pain/fatigue, and mental health. To avoid circularity in models where mental-health comorbidity was the outcome, complex multimorbidity was operationalized using only non-mental-health organ systems in those models. Mental-health comorbidity (depression, anxiety, bipolar disorder, eating disorders, insomnia, substance abuse, attention-deficit/hyperactivity disorder, autism spectrum disorders, dissociative disorders, conversion disorders, phobias, and post-traumatic stress disorder) was treated as a separate binary domain (≥ 1 vs. none). Autoimmune diseases other than IIM were captured separately to derive autoimmune multimorbidity.

### Patient-reported outcome measures

Several patient-reported outcome measures (PROMs) were collected and used in the analytic models. These included fatigue and pain visual analogue scales (VAS, 0–10), the PROMIS Global Mental Health (GMH) T-score, the PROMIS Physical Function-4a (PF-4a) short form, the Satisfaction With Life Scale (SWLS) total score, and the family APGAR score. Full definitions and scoring are detailed in the survey instrument description [16–18]. PROMs were not included in the operational definitions of the multimorbidity constructs themselves, but were examined as correlates of comorbidity burden and multimorbidity patterns.

### Statistical analysis

Continuous variables were summarized as mean ± standard deviation (SD) or median (IQR, interquartile 25th–75th), and categorical variables as frequencies and percentages (%). Group comparisons used Student’s *t*-test or the Mann–Whitney U test for continuous variables and the chi-square test and Fisher’s exact test for categorical variables.

To identify independent associations with mental-health and physical-health comorbidity, we employed a hierarchical multivariable logistic regression strategy. Two binary outcomes were modelled: (i) presence of ≥ 1 mental-health comorbidity and (ii) presence of ≥ 1 physical-health comorbidity. For each outcome, three domain-specific models were first constructed: Model 1 included socio-demographic factors (age, sex, ethnicity); Model 2 included disease-related factors (IIM subtype, disease duration, disease activity status, GC dose > 10 mg/day, current immunosuppressive therapy, and complex multimorbidity); and Model 3 included socio-economic and lifestyle factors (HDI category, educational attainment, smoking status, alcohol use, and family APGAR score). In these models, complex multimorbidity was defined as ≥ 3 affected non-mental-health organ systems, as detailed above, to minimize conceptual overlap with mental-health comorbidity.

Variables that were clinically relevant a priori or reached *p* < 0.20 in domain-specific models (Models 1–3) were then entered into a fully adjusted integrated model (Model 5). A separate exploratory model (Model 4) additionally incorporated patient-reported outcome variables (fatigue VAS, pain VAS, PROMIS GMH T-score, PROMIS PF-4a, and SWLS total score) to examine whether PROMs were independently associated with comorbidity status.

Collinearity was assessed using variance inflation factors (VIF); all retained factors had VIF < 3, indicating acceptable independence. Results are reported as adjusted odds ratios (OR) with 95% confidence intervals (CI).

Missing data were quantified for every analysis variable and are reported in full in Online Resource 2. Missingness was low for most variables (< 4%), including IIM subtype (3.7%, n = 38). Two variables exceeded this: education (5.0%, n = 51, reflecting “Prefer not to disclose” responses) and ILD status (12.4%, n = 127, comprising 72 non-responses and 55 “I am not sure” responses). Each model was fitted using complete-case analysis restricted to its included variables, and the sample contributing to each model is reported in Tables 3 and 4. Multiple imputation was not employed because missingness was low for the covariates entered into the regression models, several key factors were complete by design, and the aims of these analyses were exploratory rather than predictive.

Thirteen binary comorbidity domains (respiratory, renal, hepatobiliary, gastrointestinal, reproductive, special sensory, musculoskeletal, cardiovascular, central nervous system, endocrinological, neoplastic, chronic pain/fatigue, and mental health), age, and disease duration were standardised to zero mean and unit variance, and principal component analysis was used for dimensionality reduction; three components were retained on the basis of the scree-plot elbow, accounting for 32.9% of the total variance. Hierarchical clustering with Ward’s linkage was then applied to the retained components (Online resource 3).

The number of clusters was examined across k = 2 to 6 using the mean silhouette coefficient. The silhouette coefficient was maximised at k = 2 (0.338), but the three-cluster solution (0.285) was retained because it is a nested subdivision of the two-cluster solution that separates an elderly, IBM-predominant, malignancy-enriched group otherwise subsumed within the low-burden cluster; this choice is interpretive rather than statistical. Cluster stability was assessed by non-parametric bootstrap (200 resamples) using the Jaccard similarity coefficient.

To assess whether age and disease duration unduly determined the cluster structure, we quantified the between-cluster variance explained by each clustering variable (η^2^) and repeated the clustering using the 13 comorbidity domains alone, comparing the resulting partition with the primary solution by the adjusted Rand index.

All tests were two-sided, and differences yielding a *p* < 0.05 were considered statistically significant. Multiple comparisons involving patient characteristics were adjusted using the Benjamini-Hochberg (BH) correction for false discovery rate. BH correction was applied within each analysis rather than across the full set of exploratory comparisons conducted in this study; findings from secondary and exploratory analyses should therefore be interpreted as hypothesis-generating. Statistical analyses were performed using R (version 4.5.1) and Jamovi (version 2.7.6.0).

### Ethical considerations

The study was conducted in accordance with the principles of the Declaration of Helsinki. Ethical approval was obtained from the Institutional Ethics Committee of Sanjay Gandhi Postgraduate Institute of Medical Sciences (IEC SGPGIMS), Lucknow, India (approval date: 18 October 2023; protocol number: 2023–209-EMP-13), and from the ethics committees of all other participating institutions, where required. Electronic informed consent was obtained from all participants at the beginning of the online survey before they were permitted to participate.

## Results

### Study population and baseline characteristics

A total of 1028 adults with specialist-confirmed IIM were included in the analysis (Online Resource 1) of the 14,384 survey respondents. The median age was 59.0 years (IQR 46.0–70.0), 71.3% were female, and the majority (83.0%) resided in very-high-HDI countries (Table 1). At the time of survey completion, 47.4% reported active disease, 11.8% were receiving a GC dose > 10 mg/day, and most were on at least one immunosuppressive agent.

**Table 1:** Baseline demographic, socioeconomic, disease-related, and comorbidities of the study cohort

Variables	Total (n = 1028)	PM^p^(n = 141)	DM^e^(n = 265)	IBM^l^(n = 283)	IMNM^m^(n = 58)	ASyS^b^(n = 140)	OM^o^(n = 84)	JDM^n^(n = 19)	*p*-value (FDR BH^g^)
Demographic variables									
Age (years)	59.0 (46.0–70.0)	58.0 (48.0–66.0)	56.0 (46.0–65.0)	72.0 (66.0–76.0)	58.5 (49.0–66.8)	49.0 (43.0–60.0)	46.0 (37.8–59.2)	26.0 (17.5–38.0)	< 0.001
Sex, n (%)									
Female	733 (71.3)	122 (86.5)	230 (86.8)	137 (48.4)	48 (82.8)	87 (62.1)	77 (91.7)	13 (68.4)	< 0.001
Male	288 (28.0)	17 (12.1)	33 (12.5)	145 (51.2)	10 (17.2)	52 (37.1)	7 (8.3)	6 (31.6)	
Ethnicity, n (%)									
Caucasian (White)	759 (73.8)	103 (73.0)	211 (79.6)	264 (93.3)	42 (72.4)	69 (49.3)	33 (39.3)	11 (57.9)	< 0.001
Asian	145 (14.1)	10 (7.1)	30 (11.3)	2 (0.7)	3 (5.2)	62 (44.3)	28 (33.3)	5 (26.3)	
Hispanic	16 (1.6)	2 (1.4)	4 (1.5)	3 (1.1)	3 (5.2)	1 (0.7)	1 (1.2)	1 (5.3)	
African	14 (1.4)	3 (2.1)	0	1 (0.4)	0	3 (2.1)	6 (7.1)	1 (5.3)	
Mixed	30 (2.9)	5 (3.5)	9 (3.4)	5 (1.8)	3 (5.2)	1 (0.7)	5 (6.0)	0	
Others	38 (3.7)	12 (8.5)	8 (3.0)	4 (1.4)	5 (8.6)	4 (2.9)	4 (4.8)	0	
*Socioeconomic variables*									
HDI^k^ of country, n (%)									
Very high	853 (83.0)	131 (92.9)	234 (88.3)	281 (99.3)	50 (86.2)	74 (52.9)	44 (52.4)	16 (84.2)	< 0.001
High	63 (6.1)	4 (2.8)	17 (6.4)	2 (0.7)	7 (12.1)	7 (5.0)	24 (28.6)	0	
Medium/Low	93 (9.0)	5 (3.5)	14 (5.3)	0	0	59 (42.1)	11 (13.1)	3 (15.8)	
Smoking, n (%)									
Non-smoker	722 (70.2)	98 (69.5)	203 (76.6)	206 (72.8)	37 (63.8)	69 (49.3)	64 (76.2)	15 (78.9)	< 0.001
Ex-smoker	260 (25.3)	34 (24.1)	54 (20.4)	69 (24.4)	17 (29.3)	65 (46.4)	12 (14.3)	1 (5.3)	
Smoker	45 (4.4)	9 (6.4)	8 (3.0)	8 (2.8)	4 (6.9)	6 (4.3)	8 (9.5)	0	
Alcohol intake, n (%)									
Do not consume alcohol	412 (40.1)	57 (40.4)	123 (46.4)	86 (30.4)	26 (44.8)	35 (25.0)	54 (64.3)	11 (57.9)	< 0.001
Once a month or less	313 (30.4)	50 (35.5)	72 (27.2)	75 (26.5)	13 (22.4)	68 (48.6)	17 (20.2)	3 (15.8)	
2–4 times a month	151 (14.7)	16 (11.3)	41 (15.5)	47 (16.6)	11 (19.0)	23 (16.4)	7 (8.3)	1 (5.3)	
2–3 times a week	151 (14.7)	18 (12.8)	29 (10.9)	75 (26.5)	8 (13.8)	14 (10.0)	6 (7.1)	1 (5.3)	
Education, n (%)									
Secondary or less	341 (33.2)	54 (38.3)	73 (27.5)	113 (39.9)	21 (36.2)	34 (24.3)	28 (33.3)	8 (42.1)	0.008
Undergraduates	374 (36.4)	43 (30.5)	117 (44.2)	88 (31.1)	23 (39.7)	55 (39.3)	28 (33.3)	9 (47.4)	
Graduates	258 (25.1)	44 (31.2)	67 (25.3)	76 (26.9)	12 (20.7)	21 (15.0)	25 (29.8)	1 (5.3)	
Disease-related variables									
Disease duration (years)	7.0 (3.0–15.0)	8.0 (4.0–13.0)	5.0 (2.0–15.0)	8.0 (4.0–15.0)	4.0 (1.0–7.0)	8.0 (2.0–16.2)	9.5 (3.0–20.0)	13.0 (6.0–24.0)	< 0.001
Age at diagnosis (years)	50.0 (35.0–60.0)	49.0 (37.0–57.0)	45.0 (34.0–57.0)	61.0 (52.0–68.0)	53.0 (43.0–60.0)	41.0 (29.8–53.0)	32.5 (25.0–45.0)	7.0 (4.0–10.0)	< 0.001
Disease status, n (%)									
Active disease	487 (47.4)	82 (58.2)	125 (47.2)	107 (37.8)	33 (56.9)	76 (54.3)	39 (46.4)	5 (26.3)	< 0.001
VAS-pain (0–10)	4.0 (2.0–6.0)	5.0 (3.0–7.0)	5.0 (3.0–6.0)	3.0 (1.0–5.5)	4.0 (1.0–5.8)	4.0 (3.0–5.0)	6.0 (4.0–8.0)	3.0 (1.0–6.0)	< 0.001
Medication(s), n (%)									
GC^h^ > 10 mg/day	121 (11.8)	24 (17.0)	42 (15.8)	12 (4.2)	11 (19.0)	21 (15.0)	7 (8.3)	0	< 0.001
cs^d^DMARDs^f^	532 (51.8)	110 (78.0)	192 (72.5)	49 (17.3)	42 (72.4)	67 (47.9)	43 (51.2)	9 (47.4)	< 0.001
b^c^DMARDs	269 (26.2)	54 (38.3)	86 (32.5)	30 (10.6)	29 (50.0)	41 (29.3)	13 (15.5)	7 (36.8)	< 0.001
ts^t^DMARDs	34 (3.3)	2 (1.4)	20 (7.5)	0	0	7 (5.0)	3 (3.6)	0	< 0.001
Health-related variables									
PROMIS^r^ GPH^i^	37.6 (30.5–44.7)	37.6 (30.5–44.7)	41.2 (30.5–48.3)	37.6 (30.5–41.2)	34.0 (26.9–47.4)	41.2 (34.0–48.3)	34.0 (30.5–44.7)	48.3 (41.2–50.8)	< 0.001
PROMIS GMH^j^	52.0 (43.2–56.4)	47.6 (43.2–56.4)	47.6 (43.2–56.4)	52.0 (43.2–56.4)	52.0 (43.2–56.4)	52.0 (43.2–56.4)	47.6 (43.2–56.4)	52.0 (47.6–58.8)	0.203
PROMIS PF-4a^q^	36.0 (32.0–41.0)	37.0 (33.0–42.0)	39.0 (34.0–44.0)	32.0 (27.0–36.0)	36.0 (32.0–41.8)	37.0 (34.0–41.0)	38.0 (34.0–46.0)	40.0 (36.8–44.0)	< 0.001
SWLS^s^	18.0 (13.0–24.0)	17.0 (13.0–24.0)	17.0 (12.0–25.0)	18.0 (13.0–23.0)	19.5 (11.2–24.0)	17.5 (14.0–22.0)	17.0 (13.0–22.0)	18.0 (15.0–24.0)	0.811
Family APGAR^a^	9.0 (6.0–10.0)	8.0 (5.0–10.0)	9.0 (6.0–10.0)	9.0 (7.0–10.0)	9.0 (7.0–10.0)	7.0 (5.0–10.0)	7.0 (4.0–10.0)	8.0 (7.0–9.8)	< 0.001
Brief resilience scale	3.3 (2.8–4.0)	3.3 (2.7–4.0)	3.3 (2.7–3.8)	3.7 (3.0–4.0)	3.6 (3.0–4.0)	3.0 (2.7–3.5)	3.0 (2.5–3.7)	3.5 (3.0–4.0)	< 0.001
Comorbidities, n (%)									
Single physical comorbidity	243 (23.6)	36 (25.5)	48 (18.1)	78 (27.6)	12 (20.7)	35 (25.0)	24 (28.6)	1 (5.3)	0.086
Basic multimorbidity	480 (46.7)	71 (50.4)	133 (50.2)	148 (52.3)	28 (48.3)	34 (24.3)	44 (52.4)	8 (42.1)	< 0.001
Complex multimorbidity	158 (15.4)	26 (18.4)	50 (18.9)	40 (14.1)	10 (17.2)	9 (6.4)	13 (15.5)	5 (26.3)	0.040
Mental health comorbidity	345 (33.6)	53 (37.6)	104 (39.2)	72 (25.4)	23 (39.7)	36 (25.7)	38 (45.2)	7 (36.8)	< 0.001
Autoimmune multimorbidity	151 (14.7)	41 (29.1)	42 (15.8)	18 (6.4)	7 (12.1)	4 (2.9)	38 (45.2)	0	< 0.001
VAS^u^-fatigue (0–10)	7.0 (5.0–8.0)	7.0 (5.0–8.0)	7.0 (5.0–8.0)	7.0 (5.0–8.0)	7.0 (4.2–8.0)	5.0 (3.8–7.0)	7.0 (5.0–8.0)	5.0 (3.0–6.0)	< 0.001

Data are expressed as median (interquartile 25th—75th), or frequency (%). Percentages are based on available data and may not sum to 100% due to missing responses and rounding

^a^APGAR: Adaptation Partnership Growth Affection Resolve, ^b^ASyS: anti-synthetase syndrome, ^c^b; biological, ^d^cs: conventional synthetic, ^e^DM: dermatomyositis, ^f^DMARDs: disease-modifying antirheumatic drugs, ^g^FDR BH: false discovery rate, Benjamini–Hochberg correction, ^h^GC: glucocorticoid, ^i^GPH: Global Physical Health; ^j^GMH: Global Mental Health, ^k^HDI: Human Development Index, ^l^IBM: inclusion body myositis, ^m^IMNM: immune-mediated necrotizing myopathy, ^n^JDM: juvenile dermatomyositis, ^o^OM: overlap myositis, ^p^PM: polymyositis, ^q^PF-4a: Physical Function 4a, ^r^PROMIS: Patient-Reported Outcomes Measurement Information System, ^s^SWLS: Satisfaction With Life Scale, ^t^ts: targeted synthetic, ^u^VAS: visual analogue scale

IBM was the most frequent diagnosis, followed by DM, PM, ASyS, OM, IMNM, and JDM. Subtype totals do not sum to the full cohort (n = 990 classified across the seven subtypes) as 38 participants (3.7%) had missing IIM subtype data and were excluded from subtype-stratified analyses **(**Table 2**)** while remaining eligible for analyses not requiring subtype classification. Median age differed markedly across subtypes, ranging from 46 years in OM to 72 years in IBM. Disease duration was the longest in OM and the shortest in IMNM.

**Table 2:** Prevalence of comorbidities across idiopathic inflammatory myopathy subtypes

Variables	Total (n = 1028)	PM^s^(n = 141)	DM^i^(n = 265)	IBM^l^(n = 283)	IMNM^n^(n = 58)	ASyS^c^(n = 140)	OM^q^(n = 84)	JDM^o^(n = 19)	*p*-value (FDR BH^a^)
Respiratory									
Asthma	133 (12.9)	13 (9.2)	40 (15.1)	37 (13.1)	4 (6.9)	13 (9.3)	15 (17.9)	5 (26.3)	0.108
COPD^g^	41 (4.0)	6 (4.3)	7 (2.6)	13 (4.6)	5 (8.6)	7 (5.0)	3 (3.6)	0	0.496
ILD^m^	240 (23.3)	60 (42.6)	73 (27.5)	11 (3.9)	7 (12.1)	71 (50.7)	14 (16.7)	0	< 0.001
Renal (CKD^f^)	33 (3.2)	3 (2.1)	12 (4.5)	7 (2.5)	0	2 (1.4)	6 (7.1)	0	0.108
Liver disease	44 (4.3)	9 (6.4)	13 (4.9)	8 (2.8)	4 (6.9)	4 (2.9)	6 (7.1)	0	0.376
Gastrointestinal									
Peptic ulcer disease	23 (2.2)	5 (3.5)	5 (1.9)	3 (1.1)	0	2 (1.4)	6 (7.1)	0	0.030
Upper GI^j^ disease	188 (18.3)	30 (21.3)	59 (22.3)	46 (16.3)	10 (17.2)	13 (9.3)	21 (25.0)	3 (15.8)	0.036
Infertility	28 (2.7)	1 (0.7)	15 (5.7)	5 (1.8)	0	2 (1.4)	3 (3.6)	0	0.027
Visual impairment	165 (16.1)	22 (15.6)	42 (15.8)	60 (21.2)	4 (6.9)	6 (4.3)	20 (23.8)	4 (21.1)	< 0.001
Musculoskeletal									
Degenerative disc disease	147 (14.3)	31 (22.0)	40 (15.1)	40 (14.1)	5 (8.6)	11 (7.9)	14 (16.7)	0	0.019
Osteoporosis	154 (15.0)	17 (12.1)	53 (20.0)	39 (13.8)	10 (17.2)	9 (6.4)	15 (17.9)	5 (26.3)	0.019
Cardiovascular									
Hypertension	278 (27.0)	34 (24.1)	70 (26.4)	107 (37.8)	14 (24.1)	19 (13.6)	20 (23.8)	4 (21.1)	< 0.001
Angina	36 (3.5)	6 (4.3)	5 (1.9)	12 (4.2)	3 (5.2)	6 (4.3)	2 (2.4)	1 (5.3)	0.688
CHF^e^	48 (4.7)	5 (3.5)	7 (2.6)	19 (6.7)	6 (10.3)	5 (3.6)	4 (4.8)	1 (5.3)	0.158
Heart attack	39 (3.8)	3 (2.1)	4 (1.5)	24 (8.5)	3 (5.2)	2 (1.4)	1 (1.2)	1 (5.3)	< 0.001
PVD^u^	14 (1.4)	1 (0.7)	4 (1.5)	4 (1.4)	1 (1.7)	1 (0.7)	3 (3.6)	0	0.687
Neurological									
Stroke	26 (2.5)	3 (2.1)	6 (2.3)	6 (2.1)	4 (6.9)	0	5 (6.0)	0	0.052
Others (MS^p^/PD^r^)	27 (2.6)	7 (5.0)	5 (1.9)	8 (2.8)	2 (3.4)	0	5 (6.0)	0	0.108
Endocrinological									
Diabetes mellitus	82 (8.0)	10 (7.1)	27 (10.2)	25 (8.8)	10 (17.2)	3 (2.1)	3 (3.6)	2 (10.5)	0.017
Obesity	154 (15.0)	28 (19.9)	53 (20.0)	32 (11.3)	9 (15.5)	16 (11.4)	7 (8.3)	5 (26.3)	0.019
Infections									
HIV^k^	3 (0.3)	0	0	2 (0.7)	0	1 (0.7)	0	0	0.687
Neoplastic disease	189 (18.4)	18 (12.8)	35 (13.2)	99 (35.0)	11 (19.0)	8 (5.7)	8 (9.5)	0	< 0.001
Blood	21 (2.0)	3 (2.1)	2 (0.8)	13 (4.6)	0	0	2 (2.4)	0	0.023
Breast	28 (2.7)	5 (3.5)	5 (1.9)	7 (2.5)	4 (6.9)	3 (2.1)	1 (1.2)	0	0.398
Cervix	10 (1.0)	2 (1.4)	1 (0.4)	2 (0.7)	0	0	2 (2.4)	0	0.434
Colon	17 (1.7)	1 (0.7)	2 (0.8)	12 (4.2)	0	0	2 (2.4)	0	0.019
Head neck	5 (0.5)	0	1 (0.4)	4 (1.4)	0	0	0	0	0.380
Prostate	20 (1.9)	0	1 (0.4)	17 (6.0)	1 (1.7)	0	0	0	< 0.001
Skin	87 (8.5)	9 (6.4)	16 (6.0)	50 (17.7)	3 (5.2)	3 (2.1)	3 (3.6)	0	< 0.001
Others	27 (2.6)	0	9 (3.4)	10 (3.5)	4 (6.9)	3 (2.1)	1 (1.2)	0	0.140
Chronic pain/fatigue									
Fibromyalgia	131 (12.7)	25 (17.7)	49 (18.5)	21 (7.4)	3 (5.2)	12 (8.6)	10 (11.9)	4 (21.1)	0.001
CFS^d^	62 (6.0)	10 (7.1)	27 (10.2)	6 (2.1)	5 (8.6)	3 (2.1)	6 (7.1)	4 (21.1)	< 0.001
Post-COVID-19^ h^	24 (2.3)	5 (3.5)	8 (3.0)	0	0	2 (1.4)	8 (9.5)	1 (5.3)	< 0.001
Mental health comorbidities									
Anxiety	155 (15.1)	23 (16.3)	50 (18.9)	27 (9.5)	12 (20.7)	15 (10.7)	14 (16.7)	7 (36.8)	0.006
Bipolar disorder	10 (1.0)	0	5 (1.9)	0	3 (5.2)	1 (0.7)	1 (1.2)	0	0.019
Depression	206 (20.0)	31 (22.0)	63 (23.8)	46 (16.3)	17 (29.3)	17 (12.1)	23 (27.4)	5 (26.3)	0.019
Eating disorders	23 (2.2)	1 (0.7)	8 (3.0)	4 (1.4)	3 (5.2)	0	6 (7.1)	1 (5.3)	0.010
Insomnia	108 (10.5)	18 (12.8)	35 (13.2)	16 (5.7)	4 (6.9)	9 (6.4)	22 (26.2)	1 (5.3)	< 0.001
Substance abuse	2 (0.2)	0	0	1 (0.4)	0	1 (0.7)	0	0	0.771
ADHD^b^	22 (2.1)	1 (0.7)	10 (3.8)	3 (1.1)	1 (1.7)	2 (1.4)	2 (2.4)	3 (15.8)	0.002
Autism	7 (0.7)	0	3 (1.1)	0	0	1 (0.7)	0	2 (10.5)	< 0.001
Dissociative disorders	3 (0.3)	0	2 (0.8)	0	1 (1.7)	0	0	0	0.294
Conversion disorder	13 (1.3)	0	5 (1.9)	1 (0.4)	1 (1.7)	1 (0.7)	3 (3.6)	2 (10.5)	0.004
Phobia	12 (1.2)	4 (2.8)	3 (1.1)	0	0	0	5 (6.0)	0	0.001
PTSD^t^	53 (5.2)	10 (7.1)	19 (7.2)	8 (2.8)	3 (5.2)	5 (3.6)	2 (2.4)	3 (15.8)	0.054

Data are expressed as frequency (%). Percentages are based on available data and may not sum to 100% due to missing responses and rounding

^a^FDR BH: false discovery rate, Benjamini–Hochberg correction, ^b^ADHD: attention deficit/hyperactivity disorder, ^c^ASyS: anti-synthetase syndrome, ^d^CFS: chronic fatigue syndrome, ^e^CHF: congestive heart failure, ^f^CKD: chronic kidney disease, ^g^COPD: chronic obstructive pulmonary disease, ^h^COVID-19: coronavirus disease 2019, ^i^DM: dermatomyositis, ^j^GI: gastrointestinal, ^k^HIV: human immunodeficiency virus, ^l^IBM: inclusion body myositis, ^m^ILD: interstitial lung disease, ^n^IMNM: immune-mediated necrotizing myopathy, ^o^JDM: juvenile dermatomyositis, ^p^MS: multiple sclerosis; ^q^OM: overlap myositis, ^r^PD: Parkinson disease, ^s^PM: polymyositis, ^t^PTSD: post-traumatic stress disorder, ^u^PVD: peripheral vascular disease

### Comorbidity burden and distribution across IIM subtypes

Overall, 46.7% of patients met criteria for basic multimorbidity, 15.4% for complex multimorbidity, 33.6% had at least one mental-health comorbidity, and 14.7% had autoimmune multimorbidity. Comorbidity patterns varied markedly by IIM subtype **(**Table 2**)**: ILD concentrated in ASyS (50.7%) and PM (42.6%) but was rare in IBM (3.9%), while cardiovascular disease and malignancy concentrated in IBM (hypertension 37.8%, malignancy 35.0%), consistent with its older age profile.

Mental-health disorders were common across all subtypes but distinctly distributed, concentrating most heavily in IMNM and OM **(**Table 2**)**. Overall, younger and female patients reported higher rates of mental-health comorbidity, whereas older age was associated with a greater burden of physical comorbidities. These patterns represent cross-sectional associations and do not imply causal direction.

### Associations of mental-health comorbidity

In the fully adjusted model for mental-health comorbidity (Model 5, n = 931), complex multimorbidity remained strongly associated (OR 2.85, 95% CI 1.96–4.14, adjusted p < 0.001), as did younger age (OR 0.98 per year, 0.97–0.99, adjusted p = 0.014), immunosuppressant use (OR 1.53, 1.09–2.15, adjusted p = 0.049) and a lower family APGAR score (OR 0.94 per unit, 0.90–0.99, adjusted p = 0.049). Three associations that reached nominal significance did not survive adjustment: female sex (OR 1.48, 1.02–2.14; p = 0.040, adjusted p = 0.093), alcohol use (OR 0.72, 0.54–0.96; p = 0.023, adjusted p = 0.064) and undergraduate or higher education (OR 1.34, 1.00–1.80; p = 0.049, adjusted p = 0.098) (Table 3).

**Table 3:** Associations of mental-health comorbidities in idiopathic inflammatory myopathies (hierarchical multiple logistic regression)

Factor	OR (95% CI^q^)	p-value	Adjusted p (BH^r^)
Model 1-socio-demographic (n = 1020)			
Age (per year)	0.98 (0.98–0.99)	0.002	0.003
Female sex	1.93 (1.38–2.70)	< 0.001	< 0.001
Ethnicity (others vs. caucasian)	0.70 (0.50–0.97)	0.032	0.032
Model 2-disease-related (n = 990)			
Disease duration (years)	1.00 (0.99–1.01)	0.755	0.862
IIM^h^ subtype (reference DM^c^)			
ASyS^b^	0.68 (0.43–1.09)	0.109	0.287
IBM^g^	0.74 (0.49–1.12)	0.151	0.302
IMNM^i^	1.00 (0.55–1.82)	0.998	0.998
OM^k^	1.51 (0.90–2.54)	0.115	0.287
PM^m^	0.90 (0.59–1.39)	0.637	0.862
Complex multimorbidity	2.77 (1.93–3.97)	< 0.001	< 0.001
Active disease	1.10 (0.83–1.47)	0.500	0.833
GC^e^ > 10 mg/day	0.94 (0.61–1.45)	0.776	0.862
IS^j^	1.75 (1.26–2.43)	< 0.001	< 0.001
Model 3-socio-economic (n = 971)			
Smoking (ever)	1.14 (0.85–1.54)	0.376	0.376
Alcohol use	0.60 (0.46–0.79)	< 0.001	< 0.001
Very high HDI^d^	1.39 (0.96–2.02)	0.078	0.097
Undergraduate and above education	1.43 (1.08–1.90)	0.012	0.020
Family APGAR (per unit)	0.93 (0.89–0.98)	0.002	0.005
Model 4-health outcomes (n = 1,028)			
SWLS^o^ total	1.03 (1.01–1.06)	0.007	0.009
Fatigue VAS^p^	1.19 (1.10–1.28)	< 0.001	< 0.001
Pain VAS^p^	1.13 (1.06–1.21)	< 0.001	< 0.001
PROMIS^n^ GMH^f^ T-score	0.96 (0.94–0.97)	< 0.001	< 0.001
PROMIS PF-4a^l^	1.02 (0.99–1.05)	0.191	0.191
Model 5-fully adjusted integrated (n = 931)			
Age (per year)	0.98 (0.97–0.99)	0.002	0.014
Female sex	1.48 (1.02–2.14)	0.040	0.093
Ethnicity (others vs. caucasian)	0.78 (0.51–1.19)	0.242	0.397
IIM^h^ subtype (reference DM^c^)			
ASyS^b^	0.83 (0.51–1.35)	0.448	0.627
IBM^g^	1.13 (0.71–1.80)	0.605	0.652
IMNM^i^	1.18 (0.64–2.16)	0.601	0.652
OM^k^	1.38 (0.79–2.39)	0.255	0.397
PM^m^	0.96 (0.61–1.50)	0.847	0.847
Complex multimorbidity	2.85 (1.96–4.14)	< 0.001	< 0.001
IS^j^	1.53 (1.09–2.15)	0.014	0.049
Alcohol use	0.72 (0.54–0.96)	0.023	0.064
Very high HDI^d^	1.20 (0.71–2.03)	0.496	0.631
Undergraduate and above education	1.34 (1.00–1.80)	0.049	0.098
Family APGAR^a^ (per unit)	0.94 (0.90–0.99)	0.011	0.049

^a^APGAR: Adaptation Partnership Growth Affection Resolve, ^b^ASyS: anti-synthetase syndrome, ^c^DM: dermatomyositis, ^d^HDI: Human Development Index, ^e^GC: glucocorticoid, ^f^GMH: Global Mental Health, ^g^IBM: inclusion body myositis, ^h^IIM: idiopathic inflammatory myopathies, ^i^IMNM: immune-mediated necrotizing myopathy, ^j^IS: immunosuppressive, ^k^OM: overlap myositis, ^l^PF-4a: Physical Function 4a, ^m^PM: polymyositis, ^n^PROMIS: Patient-Reported Outcomes Measurement Information System, ^o^SWLS: Satisfaction With Life Scale, ^p^VAS: visual analogue scale, ^q^CI, confidence interval, ^r^BH, Benjamini-Hochberg

To identify associations of comorbidities, a hierarchical multiple logistic regression approach was employed. First, domain-specific models were constructed to assess the variance explained by distinct categories of factors: (1) sociodemographic factors, (2) disease-specific factors, and (3) socioeconomic models. Clinically relevant variables and variables with *p* < 0.20 in domain specific variables were combined to form a fully adjusted integrated model. A separate model was constructed to include health outcomes. Model 1 (socio-demographic), Model 2 (disease-related), Model 3 (socio-economic), Model 4 (health-outcome), and Model 5 (fully adjusted integrated). Model 4 includes health-outcome variables (fatigue VAS, pain VAS, PROMIS GMH, PROMIS PF-4a, SWLS)

Multicollinearity was assessed using variance inflation factors (VIF), with VIF < 3 indicating acceptable independence. Results were reported as adjusted odds ratios (ORs), 95%CIs, and adjusted *p*-values

^*^p < 0.05, **p < 0.001

### Associations of physical-health comorbidity

In the fully adjusted model for physical-health comorbidity (Model 5, n = 976, Table 4), mental-health comorbidity (OR 3.96, 2.72–5.77), older age (OR 1.05 per year, 1.03–1.06) and overlap myositis (OR 3.33, 1.65–6.75) all remained significant after adjustment (all adjusted p < 0.001). In the disease-related model (Model 2, n = 990), the associations with inclusion body myositis (OR 2.46, 1.56–3.87, adjusted p < 0.001), mental-health comorbidity (OR 3.37, 2.38–4.78, adjusted p < 0.001), longer disease duration (OR 1.02 per year, 1.01–1.04, adjusted p = 0.007) and ASyS (OR 0.54, 0.34–0.85, adjusted p = 0.017) persisted, whereas OM (p = 0.043, adjusted p = 0.072) and immunosuppressant use (p = 0.041, adjusted p = 0.072) did not. In the socio-economic model (Model 3, n = 971), alcohol use (OR 0.60, 0.45–0.81) and residence in a very high HDI country (OR 2.78, 1.95–3.97) remained significant (both adjusted p < 0.001), while caring responsibilities did not (p = 0.047, adjusted p = 0.094).

**Table 4:** Associations of physical-health comorbidities in idiopathic inflammatory myopathies (hierarchical multiple logistic regression)

Factor	OR (95% CI^q^)	p-value	Adjusted p (BH^r^)
Model 1-Socio-demographic (n = 1020)			
Age (per year)	1.04 (1.03–1.05)	< 0.001	< 0.001
Sex (female vs. male)	1.80 (1.29–2.50)	< 0.001	< 0.001
Ethnicity (others vs. caucasian)	0.73 (0.52–1.01)	0.056	0.056
Model 2-disease-related (n = 990)			
Disease duration (years)	1.02 (1.01–1.04)	0.002	0.007
ASyS^b^	0.54 (0.34–0.85)	0.007	0.017
IBM^g^	2.46 (1.56–3.87)	< 0.001	< 0.001
IMNM^i^	1.10 (0.58–2.10)	0.770	0.770
OM^k^	1.93 (1.02–3.64)	0.043	0.072
PM^m^	1.46 (0.90–2.37)	0.123	0.154
Active disease	0.89 (0.66–1.21)	0.471	0.523
GC^e^ > 10 mg/day	0.68 (0.43–1.07)	0.091	0.130
IS^j^	1.45 (1.02–2.07)	0.041	0.072
Mental health comorbidity	3.37 (2.38–4.78)	< 0.001	< 0.001
Model 3-socio-economic (n = 971)			
Smoking (ever)	1.05 (0.77–1.43)	0.758	0.873
Alcohol use	0.60 (0.45–0.81)	< 0.001	< 0.001
Very high HDI^d^	2.78 (1.95–3.97)	< 0.001	< 0.001
Undergraduate and above education	0.90 (0.67–1.20)	0.458	0.687
Family APGAR^a^ (per unit)	1.00 (0.95–1.05)	0.873	0.873
Caring responsibilities	0.73 (0.54–0.99)	0.047	0.094
Model 4-health outcomes (n = 1028)			
SWLS^o^ total	1.00 (0.98–1.02)	0.932	0.932
Fatigue VAS^p^	1.14 (1.06–1.22)	< 0.001	< 0.001
Pain VAS^p^	1.02 (0.95–1.09)	0.625	0.781
PROMIS^n^ GMH^f^ T-score	1.02 (1.01–1.04)	0.023	0.038
PROMIS^n^ PF-4a^l^	0.90 (0.87–0.93)	< 0.001	< 0.001
Model 5-Fully adjusted integrated (n = 976)			
Age (per year)	1.05 (1.03–1.06)	< 0.001	< 0.001
Sex (female vs. male)	1.27 (0.86–1.86)	0.228	0.342
Ethnicity (others vs. Caucasian)	0.71 (0.45–1.11)	0.135	0.317
Disease duration (years)	1.02 (1.00–1.03)	0.053	0.199
ASyS^b^	0.73 (0.44–1.21)	0.218	0.342
IBM^g^	1.13 (0.67–1.90)	0.657	0.817
IMNM^i^	0.90 (0.46–1.77)	0.766	0.821
OM^k^	3.33 (1.65–6.75)	< 0.001	< 0.001
PM^m^	1.43 (0.86–2.40)	0.169	0.317
GC^e^ > 10 mg/day	0.72 (0.46–1.14)	0.164	0.317
IS^j^	1.13 (0.77–1.66)	0.537	0.732
Mental health comorbidity	3.96 (2.72–5.77)	< 0.001	< 0.001
Alcohol use	0.74 (0.53–1.03)	0.074	0.222
Very high HDI^d^	0.90 (0.52–1.56)	0.708	0.817
Caring responsibilities	0.99 (0.70–1.40)	0.950	0.950

^a^APGAR: Adaptation Partnership Growth Affection Resolve, ^b^ASyS: anti-synthetase syndrome, ^c^DM: dermatomyositis, ^d^HDI: Human Development Index, ^e^GC: glucocorticoid, ^f^GMH: Global Mental Health, ^g^IBM: inclusion body myositis, ^h^IIM: idiopathic inflammatory myopathies, ^i^IMNM: immune-mediated necrotizing myopathy, ^j^IS: immunosuppressive, ^k^OM: overlap myositis, ^l^PF-4a: Physical Function 4a, ^m^PM: polymyositis, ^n^PROMIS: Patient-Reported Outcomes Measurement Information System, ^o^SWLS: Satisfaction With Life Scale, ^p^VAS: visual analogue scale, ^q^CI, confidence interval, ^r^BH, Benjamini-Hochberg. Hierarchical logistic regression models were constructed to identify independent factors associated with physical multimorbidity

Model 1 (socio-demographic); Model 2 (disease-related); Model 3 (socio-economic); Model 4 (health-outcome); Model 5 (fully adjusted integrated). In Model 3 (Lifestyle/Social), very high HDI appeared associated with increased physical multimorbidity (OR = 2.78); however, this association was confounded by other variables and became non-significant when adjusted for age, disease duration, mental health comorbidities, and IIM subtype in the integrated model (OR = 0.90, *p* = 0.708), indicating that HDI is not an independent association. Mental health comorbidities demonstrated the strongest independent association with physical multimorbidity (OR = 3.96, 95% CI = 2.72–5.77, *p* < 0.001). Age and OM were also robust independent associations. Results are reported as adjusted OR, 95% CI, and adjusted *p*-values. Multicollinearity was assessed using variance inflation factors < 3 indicating acceptable independence

### Chronic pain and fatigue syndromes and comorbidity patterns

Participants with chronic pain or fatigue syndromes had a substantially higher burden of comorbidities across nearly every organ system compared with those without such syndromes (Table 5). Specifically, they exhibited higher frequencies of respiratory (45.4% *vs.* 33.0%), gastrointestinal (33.3% *vs.* 16.2%), special sensory (28.7% *vs.* 13.5%), musculoskeletal (42.5% *vs.* 22.2%), cardiovascular (44.3% *vs.* 31.4%), and endocrinological comorbidities (31.0% *vs.* 17.4%; all *p* ≤ 0.005). The disparity was most striking for mental-health comorbidities (63.2% *vs.* 27.5%, *p* < 0.001), representing an approximately two-fold increase in mental-health burden among those with chronic pain or fatigue. This suggests that chronic pain and fatigue syndromes cluster with multi-organ and mental-health comorbidity in IIM, although causal direction cannot be inferred.

**Table 5:** Comparison of comorbidity frequencies in participants with and without chronic pain/fatigue syndrome

Variables	Total (n = 1028)	Chronic pain/fatigue syndrome (+) (n = 174)	Chronic pain/fatigue syndrome (-) (n = 854)	*p*-value (FDR BH^b^)
Respiratory	361 (35.1)	79 (45.4)	282 (33.0)	0.005
Renal	33 (3.2)	7 (4.0)	26 (3.0)	0.787
Hepatobiliary disease	44 (4.3)	12 (6.9)	32 (3.7)	0.138
Gastrointestinal	196 (19.1)	58 (33.3)	138 (16.2)	< 0.001
Infertility	28 (2.7)	8 (4.6)	20 (2.3)	0.206
Special sensory	165 (16.1)	50 (28.7)	115 (13.5)	< 0.001
Musculoskeletal	264 (25.7)	74 (42.5)	190 (22.2)	< 0.001
Cardiovascular	345 (33.6)	77 (44.3)	268 (31.4)	0.003
Central nervous system	26 (2.5)	9 (5.2)	17 (2.0)	0.049
Endocrinological	203 (19.7)	54 (31.0)	149 (17.4)	< 0.001
Infections (HIV^a^)	3 (0.3)	0	3 (0.4)	0.990
Neoplastic disease	189 (18.4)	30 (17.2)	159 (18.6)	0.811
Mental health comorbidities	345 (33.6)	110 (63.2)	235 (27.5)	< 0.001

Data are presented as frequency (%)

^a^HIV: human immunodeficiency virus, ^b^FDR-BH: Benjamini–Hochberg correction for false discovery rate

### Interstitial lung disease and comorbidity patterns

Stratification by ILD status revealed no major differences in the prevalence of other organ-system comorbidities (Online resource 4). Cardiovascular disease tended to be less frequent among patients with ILD than those without (25.8% *vs.* 35.9%, *p* = 0.063), whereas chronic pain and fatigue syndromes were numerically more common in the ILD group (21.2% *vs.* 15.6%, *p* = 0.218). Mental-health comorbidity prevalence did not differ between groups (36.7% *vs.* 32.6%, *p* = 0.721).

### Cluster analysis: three distinct multimorbidity profiles

**Fig. 1:**
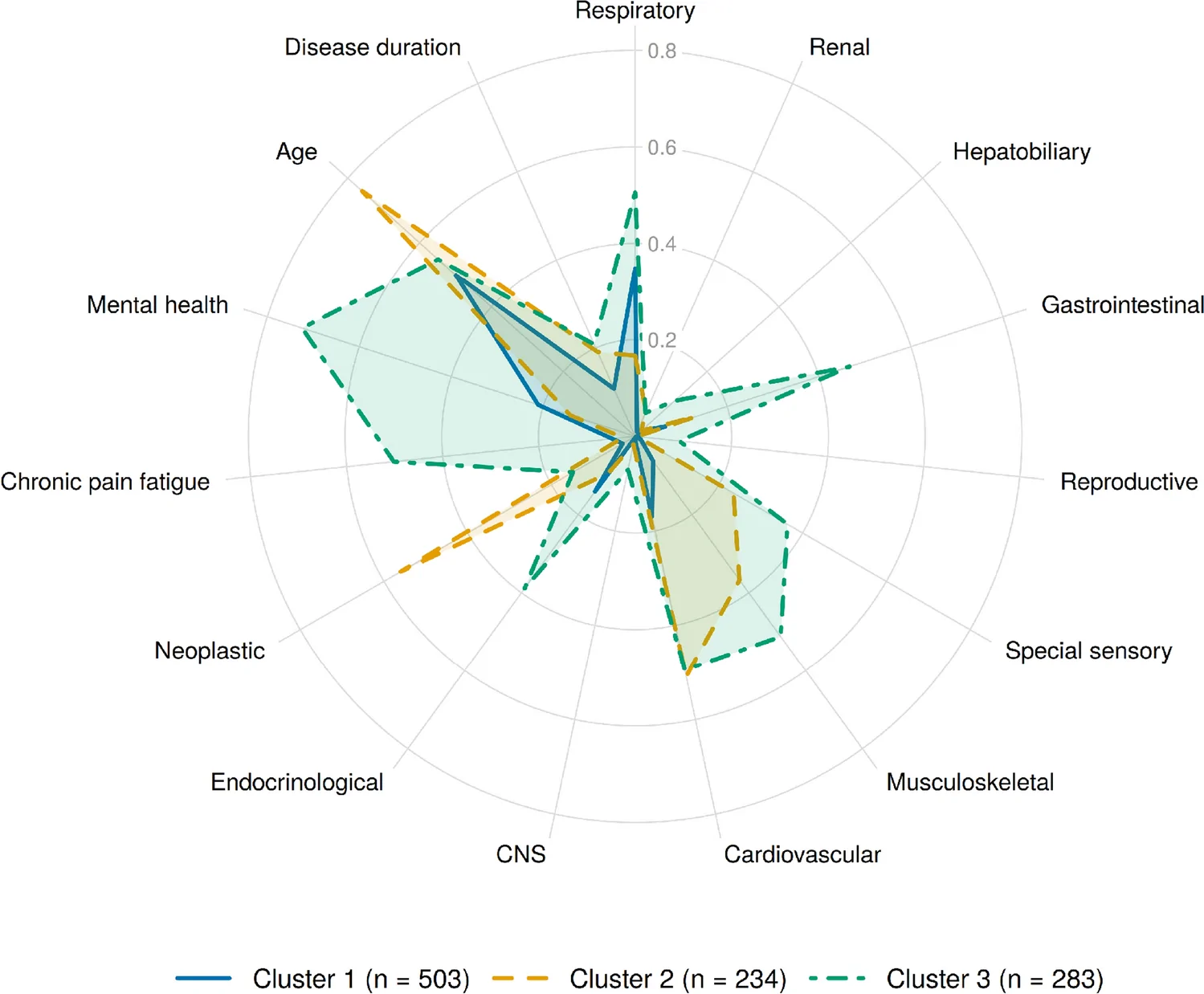
Radar plot. Multimorbidity profiles of the three clusters (n = 1020). CNS, central nervous system. Each spoke is one of the 15 variables entered into the cluster analysis, arranged clockwise from the top. For the 13 binary comorbidity domains (respiratory through mental health), radial distance is the within-cluster prevalence of that domain on a 0–1 scale; the highest is mental-health comorbidity in Cluster 3 (0.72). Age and disease duration are continuous variables and were min–max scaled onto the same axis for display only (age across 18–88 years, disease duration across 0–71 years); their radial position is a rescaled mean, not a prevalence, and is not comparable with the comorbidity spokes. The radial axis is truncated at 0.85, above the largest plotted value (age in Cluster 2, 0.76). Cluster 1 (n = 503, 49.3%), low comorbidity burden; Cluster 2 (n = 234, 22.9%), older and inclusion-body-myositis-predominant, with malignancy and cardiovascular disease; Cluster 3 (n = 283, 27.7%), pain-fatigue and mental-health burden. On bootstrap resampling (200 resamples) only Cluster 1 was reasonably stable (Jaccard 0.73; Clusters 2 and 3, 0.47 and 0.59)

Unsupervised clustering of 1,020 participants identified three multimorbidity patterns (Fig. 1, Online resource 5). Cluster 1 (n = 503, 49.3%) was a low-burden group with the youngest median age (53 years), the shortest disease duration (5 years) and a functional comorbidity index of 1.0; basic multimorbidity was present in 20.1% and complex multimorbidity in only 2.4%. Cluster 2 (n = 234, 22.9%) was the oldest group (median age 72.5 years) and IBM-predominant (60%), characterised by malignancy (56.0%), cardiovascular disease (50.9%), hypertension (41.5%) and musculoskeletal disease (36.8%), but with the lowest prevalence of mental-health comorbidity (14.1%) and of chronic pain and fatigue syndromes (2.6%) of the three clusters. Cluster 3 (n = 283, 27.7%) was DM-predominant (36%) and defined by a high burden of mental-health comorbidity (72.4%), chronic pain and fatigue syndromes (50.2%), musculoskeletal (51.2%), respiratory (50.5%) and gastrointestinal (46.6%) disease, with fibromyalgia in 38.2% and complex multimorbidity in 39.6%; this cluster reported the highest fatigue (VAS 8.0) and pain (VAS 6.0) scores and the lowest life satisfaction (SWLS 17.0). All between-cluster comparisons remained significant after BH adjustment.

Cluster separation was driven chiefly by comorbidity domains, chronic pain/fatigue syndromes (η^2^ = 0.31), neoplastic disease (0.30) and mental-health comorbidity (0.26), with age ranking fourth (0.24) and disease duration ninth (0.09) of the 15 variables (Online resource 6 and 7). Clustering on the comorbidity domains alone reproduced the same three phenotypes (80% concordance; adjusted Rand index 0.51), including the malignancy-and-cardiovascular group and the mental-health-and-pain/fatigue group, indicating that the comorbidity structure of the clusters was not an artefact of including age or disease duration.

These patterns should be interpreted as descriptive gradients of comorbidity burden rather than as discrete patient subgroups. The mean silhouette coefficient for the three-cluster solution was 0.285, indicating weak separation, and on bootstrap resampling only Cluster 1 was reasonably stable (Jaccard 0.73; Clusters 2 and 3, 0.47 and 0.59 respectively).

## Discussion

This study demonstrates that comorbidity burden in IIM is substantial, heterogeneous, and associated with demographic, disease-related, psychosocial, and socioeconomic factors, consistent with previous autoimmune disease literature [19–25]. Three distinct multimorbidity patterns via clustering further highlight the multidimensional nature of disease burden and suggest that uniform surveillance approaches may be insufficient.

Our findings extend previous work describing multimorbidity in IIM [10] by examining mental- and physical-health comorbidity separately using hierarchical multivariable regression incorporating disease-related, socioeconomic, lifestyle, and psychosocial factors. Family support remained independently associated with mental-health comorbidity, whereas educational attainment and alcohol use showed exploratory associations that did not remain significant after BH correction. Our findings are also consistent with recent clinic-based and claims-based cohorts [26, 27]. Unlike these clinician- or claims-ascertained cohorts, our multinational, patient-reported sample using a function-oriented index (FCI) captures disability-relevant comorbidity burden that mortality- or billing-code-weighted indices may underrepresent. Additionally, extending a nationwide cohort demonstrating increased psychiatric disease in PM/DM [28], we identified mental-health comorbidity as one of the strongest independent correlates of physical-health comorbidity, a relationship not previously examined in IIM. Our cluster analysis further identified multimorbidity profiles with distinct patient-reported outcome patterns, while chronic pain and fatigue syndromes emerged as important correlates of multisystem comorbidity. As an observational study, these findings should not be interpreted as causal.

Our findings also align with the broader literature on multimorbidity in IIM. The concentration of cardiovascular disease and malignancy in IBM is consistent with reports linking age-related risk accumulation and degenerative biology to these outcomes [29], while the predominance of ILD in ASyS and PM mirrors the pulmonary tropism of anti-synthetase autoimmunity [30, 31]. The strong concurrent relationship we observed between mental- and physical-health comorbidity is consistent with broader rheumatic-disease literature describing a bidirectional relationship between the central nervous and immune systems, whereby psychological stress may influence the pathophysiology of inflammatory disease [32]. Mental-health comorbidities, present in one-third of participants and especially pronounced in OM and IMNM, are consistent with earlier observations that psychological burden is frequently under-recognized in myositis [33], and with case–control data linking psychosocial factors to mental health and fatigue in this population [34, 35]. Chronic pain and fatigue syndromes emerged as important correlates of multisystem comorbidity, consistent with studies linking these conditions to systemic inflammation and psychosocial distress in IIM [36–39].

The rates of basic and complex multimorbidity in our cohort exceeded those reported in broader autoimmune disease populations [10], suggesting that IIM may be associated with a particularly high burden of multisystem involvement. The elevated mental-health comorbidity across multiple subtypes is consistent with prior calls to integrate psychological care into routine IIM management [10].

These organ-specific patterns support further evaluation of tailored surveillance strategies. The predominance of ILD in ASyS aligns with existing pulmonary monitoring recommendations [30], whereas the concentration of cardiovascular disease and malignancy in IBM supports cardiovascular risk assessment and age-appropriate cancer screening [29]. The independent association between OM and physical-health comorbidity also warrants prospective evaluation.

The three multimorbidity patterns identified by unsupervised clustering offer a complementary, data-driven perspective on disease heterogeneity. Cluster 1 (nearly half of patients) was a low-burden group with preserved physical function and low fatigue, reflecting younger age and shorter disease duration. Cluster 2, enriched for IBM and older patients, was distinguished by malignancy and cardiovascular disease, consistent with age-related biological decline [29], but paradoxically had the lowest burden of mental-health comorbidity and of chronic pain/fatigue syndromes among the three clusters. Cluster 3, enriched for DM, was distinguished not by the worst physical comorbidity burden but by the highest fatigue and pain scores and the lowest life satisfaction, underscoring that fatigue, pain, and mental-health comorbidity are closely associated with reduced well-being in this subgroup [10, 21].

Mental-health comorbidity was independently associated with younger age, complex multimorbidity, immunosuppressive therapy, and lower family support, suggesting multiple interacting correlates. The positive association with SWLS observed only in the exploratory model did not persist after full adjustment and likely reflects residual confounding or reverse causation rather than a robust relationship. The strong concurrent association between mental- and physical-health comorbidity warrants longitudinal evaluation to clarify temporal relationships.

This is among the largest patient-centred evaluations of multimorbidity across the IIM spectrum (> 1,000 participants, diverse geographic backgrounds). However, this study has several limitations. Its cross-sectional design precludes causal inference. Reliance on self-reported comorbidities is a concern: although the FCI is validated for patient-reported use, diagnoses were not verified against medical records, and misclassification risk likely varies across conditions. Self-report may under-capture subclinical disease (e.g., ILD, malignancy) and cannot always distinguish clinician-confirmed from patient-suspected diagnoses, a risk compounded for psychiatric diagnoses by variation in diagnostic thresholds and stigma across countries. Selection bias is also likely, as participants were recruited through patient organizations and online/social media platforms rather than consecutive clinic sampling, which may favor more engaged, symptomatic, or internet-literate patients and limits representativeness. This is particularly relevant to socioeconomic and geographic comparisons: 83.0% resided in very-high-HDI countries, and the HDI association with physical-health comorbidity did not persist after full adjustment, possibly reflecting differential internet access, health literacy, or patient-organization engagement rather than a true population-level difference. Residual confounding is a concern, as disease-specific factors (cumulative glucocorticoid exposure, disease severity, organ damage, autoantibody profile) were not fully captured, potentially biasing associations in either direction. Self-reported comorbidities collected via an online survey, without independent medical-record or clinician validation, further introduce recall and misclassification bias beyond what the FCI's general validation addresses (see above). The absence of detailed treatment data, including cumulative drug use, may have influenced estimates of comorbidity burden. Multiple subgroup and exploratory comparisons were performed across this study; although false discovery rate correction was applied within each individual analysis. Additionally, as a general-population instrument, the FCI's core items may not fully capture myositis-specific burden; despite supplementation with ILD and malignancy items, disease- and treatment-related comorbidity could not be distinguished, potentially overestimating disease-attributable risk. The cluster solution is exploratory and best interpreted as descriptive gradients of burden rather than discrete disease entities, warranting replication.

Despite these limitations, this study shows that multimorbidity in IIM is common and associated with biological, psychosocial, and socioeconomic forces. These findings may inform future prospective evaluation of integrated mental-health screening and subtype-tailored surveillance strategies in IIM. Future longitudinal studies should investigate whether targeting mental-health burden, chronic pain, and modifiable lifestyle factors can alter multimorbidity trajectories and improve long-term outcomes.

## Conclusion

Comorbidity in IIM is common, heterogeneous, and associated with demographic, disease-related, psychosocial, and socioeconomic factors. Nearly half of patients met criteria for basic multimorbidity, one-third reported mental-health comorbidity, and distinct subtype-specific patterns highlight the need for tailored surveillance. Mental-health comorbidity was one of the strongest independent correlates of physical-health comorbidity, while clustering identified three multimorbidity profiles with distinct patient-reported outcomes. Together, these findings provide a rationale for prospective evaluation of integrated mental-health screening, subtype-tailored surveillance, and multidisciplinary management in IIM.

## Supplementary Information

Below is the link to the electronic supplementary material.

Supplementary Material 1

## Data Availability

The data that support the findings of this study are available from the corresponding author upon reasonable request.

## Declarations

### Declarations

Preliminary findings from this study were presented, in part, as a conference abstract at American College of Rheumatology (ACR) Convergence 2025: Coskun Benlidayi I, Lama M, Afolayan O, Shah M, Sarkar M, Rubinstein T, Opinc-Rosiak A, Miskovic R, Milchert M, Luz Felipe da Silva D, Shinjo S, Nikiphorou E, Parodis I, Venerito V, Agarwal V, Gupta L. Patterns of Comorbidity in Idiopathic Inflammatory Myopathies: A Cross-sectional Analysis of Disease Burden and Risk Factors [abstract]. Arthritis Rheumatol. 2025;77 (suppl 9). Available from: https://acrabstracts.org/abstract/patterns-of-comorbidity-in-idiopathic-inflammatory-myopathies-a-cross-sectional-analysis-of-disease-burden-and-risk-factors/. Accessed July 11, 2026. No DOI is assigned to individual ACR Convergence abstracts. The present manuscript reflects an updated analysis with a refined study sample and expanded methodology. Claude AI (Anthropic large language model) was used solely to assist with spelling and grammar checks during manuscript preparation (May 2026). The authors reviewed, verified, and edited all AI-assisted suggestions and take full responsibility for the final content of the manuscript. No part of this report is copied, published elsewhere, or generated by AI. The views and opinions expressed are solely those of the author and do not represent or reflect those of any affiliated institution (LG).

## References

[1] Lundberg IE, de Visser M, Werth VP (2018) Classification of myositis. Nat Rev Rheumatol 14:269–278. 10.1038/nrrheum.2018.41 29651121

[2] Salajegheh MK, Amato AA (2025) Idiopathic inflammatory myopathies. Continuum (Minneap Minn) 31:1385–1408. 10.1212/cont.0000000000001617 41037168

[3] Wu H, Li X, Xu H, Li Z, Feng F, Zhang J et al (2025) Malignancy in idiopathic inflammatory myopathies: recent insights. Clin Rev Allergy Immunol 68:83. 10.1007/s12016-025-09080-z PMC12361316 40824431

[4] Shah M, Shinjo SK, Day J, Gupta L (2023) Cardiovascular manifestations in idiopathic inflammatory myopathies. Clin Rheumatol 42:2557–2575. 10.1007/s10067-023-06599-4 PMC10497702 37148365

[5] de Moraes MT, de Souza FH, de Barros TB, Shinjo SK (2013) Analysis of metabolic syndrome in adult dermatomyositis with a focus on cardiovascular disease. Arthritis Care Res (Hoboken) 65:793–799. 10.1002/acr.21879 23139205

[6] Sehgal S, Patel A, Chatterjee S, Fernandez AP, Farver C, Yadav R et al (2025) Idiopathic inflammatory myopathies related lung disease in adults. Lancet Respir Med 13:272–288. 10.1016/S2213-2600(24)00267-4 PMC13138733 39622261

[7] Campar A, Alves I, da Silva AM, Farinha F, Vasconcelos C (2023) Idiopathic inflammatory myopathies - the burden of disease: cohort analysis focusing on damage and comorbidities. Autoimmun Rev 22:103455. 10.1016/j.autrev.2023.103455 37778406

[8] Hysa E, Vojinovic T, Gotelli E, Alessandri E, Pizzorni C, Paolino S et al (2023) The dichotomy of glucocorticosteroid treatment in immune-inflammatory rheumatic diseases: an evidence-based perspective and insights from clinical practice. Reumatologia 61:283–293. 10.5114/reum/170845 PMC10515127 37745141

[9] DiRenzo D, Bingham CO 3rd, Mecoli CA (2019) Patient-reported outcomes in adult idiopathic inflammatory myopathies. Curr Rheumatol Rep 21:62. 10.1007/s11926-019-0862-5 PMC7050440 31741079

[10] Fornaro M, Venerito V, Pellico MR, Iannone F, Joshi M, Chen YM et al (2025) The impact of multimorbidity on quality of life in inflammatory myopathies: a cluster analysis from the COVAD dataset. Rheumatology (Oxford) 64:2133–2142. 10.1093/rheumatology/keae520 PMC11962953 39324556

[11] Kadam E, Javaid M, Sen P, Saha S, Ziade N, Day J et al (2024) Collating the voice of people with autoimmune diseases: methodology for the third phase of the COVAD studies. Rheumatol Int 44:1233–1244. 10.1007/s00296-024-05562-z PMC11178609 38609655

[12] Zimba O, Gasparyan AY (2023) Designing, conducting, and reporting survey studies: a primer for researchers. J Korean Med Sci 38(48):e403. 10.3346/jkms.2023.38.e403 PMC10713437 38084027

[13] Groll DL, To T, Bombardier C, Wright JG (2005) The development of a comorbidity index with physical function as the outcome. J Clin Epidemiol 58:595–602. 10.1016/j.jclinepi.2004.10.018 15878473

[14] World Health Organization (2016) Multimorbidity: technical series on safer primary care. World Health Organization, Geneva

[15] Marengoni A, Vetrano DL (2021) Multimorbidity: disease of society? Lancet Healthy Longev 2:e451–e452. 10.1016/S2666-7568(21)00167-7 36097992

[16] Rose M, Bjorner JB, Becker J, Fries JF, Ware JE (2008) Evaluation of a preliminary physical function item bank supported the expected advantages of the Patient-Reported Outcomes Measurement Information System (PROMIS). J Clin Epidemiol 61:17–33. 10.1016/j.jclinepi.2006.06.025 18083459

[17] Cella D, Riley W, Stone A, Rothrock N, Reeve B, Yount S et al (2010) The Patient-Reported Outcomes Measurement Information System (PROMIS) developed and tested its first wave of adult self-reported health outcome item banks: 2005-2008. J Clin Epidemiol 63:1179–1194. 10.1016/j.jclinepi.2010.04.011 PMC2965562 20685078

[18] Schalet BD, Hays RD, Jensen SE, Beaumont JL, Fries JF, Cella D (2016) Validity of PROMIS physical function measured in diverse clinical samples. J Clin Epidemiol 73:112–118. 10.1016/j.jclinepi.2015.08.039 PMC4968197 26970039

[19] Noboa MER, Tskhakaia I, Andrews JS (2026) Comorbidities in idiopathic inflammatory myopathies: population-based evidence on risk subgroups and implications for delivery of care. Curr Opin Rheumatol 38:76–82. 10.1097/BOR.0000000000001143 41355510

[20] Yoshida A, Kim M, Kuwana M, Ravichandran N, Makol A, Sen P et al (2022) Autoimmune multimorbidity and fatigue in women with idiopathic inflammatory myopathies: an international, patient-reported, e-survey. Lancet Rheumatol 4(suppl):S10–S11. 10.1016/S2665-9913(22)00162-1

[21] Yoshida A, Kim M, Kuwana M, Ravichandran N, Makol A, Sen P et al (2024) Gender differences in patient experience in idiopathic inflammatory myopathies: subanalysis from the COVAD dataset. Mod Rheumatol 34:756–766. 10.1093/mr/road094 37769200

[22] Grignaschi S, Kim M, Zanframundo G, Ravichandran N, Lilleker JB, Sen P et al (2023) High fatigue scores in patients with idiopathic inflammatory myopathies: a multigroup comparative study from the COVAD e-survey. Rheumatol Int 43:1637–1649. 10.1007/s00296-023-05344-z PMC10265550 37314497

[23] Armadans-Tremolosa I, Selva-O’Callaghan A (2023) Inflammatory myopathy in adults, health-related quality of life, and wellbeing: a round trip between immune disease and wellness. Expert Rev Clin Immunol 19:1239–1246. 10.1080/1744666X.2023.2238128 37452824

[24] Hung HM, Chen MF, Chen CH (2020) The clinically crucial predictors of depression in women with systemic autoimmune diseases. Health Care Women Int 41:293–307. 10.1080/07399332.2019.1623796 31246540

[25] Duremala F, Tiniakou E, Andrews J (2025) Epidemiology of myositis. Curr Opin Rheumatol 37:121–127. 10.1097/BOR.0000000000001076 PMC13050537 39655458

[26] Conticini E, Khursheed T, Anuja AK, Agarwal V, Gupta L (2024) Comorbidities in idiopathic inflammatory myopathies: data from the MyoCite cohort. Int J Rheum Dis 27:e14995. 10.1111/1756-185X.14995 38062892

[27] George M, Romich E, Riley TR, England BR, Daigle S, Holladay EE et al (2026) Characteristics, treatments and outcomes of patients with dermatomyositis using real-world data. RMD Open 12:e006356. 10.1136/rmdopen-2025-006356 PMC12853428 41592913

[28] Lee IP, Lee YT, Wu FY, Su CF, Kao JH, Lin SH et al (2025) Epidemiology and risk of psychiatric disorders in patients with polymyositis and dermatomyositis: a nationwide population-based cohort study in Taiwan. BMJ Open 15:e097829. 10.1136/bmjopen-2024-097829 PMC12121564 40436454

[29] Damian L, Login CC, Solomon C, Belizna C, Encica S, Urian L et al (2022) Inclusion body myositis and neoplasia: a narrative review. Int J Mol Sci 23(13):7358. 10.3390/ijms23137358 PMC9266341 35806366

[30] Patel P, Marinock JM, Ajmeri A, Brent LH (2024) A review of antisynthetase syndrome-associated interstitial lung disease. Int J Mol Sci 25:4453. 10.3390/ijms25084453 PMC11050089 38674039

[31] Ghanbar MI, Danoff SK (2024) Review of pulmonary manifestations in antisynthetase syndrome. Semin Respir Crit Care Med 45:365–385. 10.1055/s-0044-1785536 38710221

[32] Bekarissova S, Bekarisov O, Bekaryssova D (2024) An integrated approach to the treatment of rheumatic diseases: the role of psychological interventions. Rheumatol Int 44:2727–2735. 10.1007/s00296-024-05728-9 39400563

[33] Lanis A, Alexanderson H, Ardalan K, Edison S, Graham CD, de Groot I et al (2024) Mental health in paediatric and adult myositis-related diseases: current state of research, interventions, and future steps from the MIHRA Psychological Impact Scientific Working Group. Clin Exp Rheumatol 42:413–424. 10.55563/clinexprheumatol/cngdfn 38488093

[34] Gonçalves Júnior J, Dos Santos AM, Sampaio RAAF, do Nascimento Silva T, Martines G, de Araújo DB et al (2024) Spirituality, religiosity, and mental health in patients with idiopathic inflammatory myopathies: a Brazilian multicentric case-control study. Int J Environ Res Public Health 21:653. 10.3390/ijerph21060653 PMC11203193 38928900

[35] Gonçalves Júnior J, Dos Santos AM, Sampaio RA, Silva TN, Araujo D, Cândido EL et al (2025) Religiosity, spirituality, and fatigue in patients with idiopathic inflammatory myopathies: a Brazilian multicentric case-control study. Cureus 17:e86858. 10.7759/cureus.86858 PMC12296893 40718316

[36] Eccles JA, Davies KA (2021) The challenges of chronic pain and fatigue. Clin Med (Lond) 21(1):19–27. 10.7861/clinmed.2020-1009 PMC7850224 33479064

[37] Glaubitz S, Lee YC, Saygin D (2026) Pain in myositis: prevalence, assessment, potential mechanisms and considerations for management. Clin Exp Rheumatol 44:376–383. 10.55563/clinexprheumatol/4msi8i 41678188

[38] George TB, Keret S, Pillai AC, Moghadam-Kia S, Oddis CV, Dianxu R et al (2025) Fatigue is common in myositis and is associated with disease activity. Semin Arthritis Rheum 73:152730. 10.1016/j.semarthrit.2025.152730 40273743

[39] Ma AK, Dai F, Roy B (2023) In-patient comorbidities in inclusion body myositis: a United States national in-patient sample-based study. Clin Exp Rheumatol 41:261–266. 10.55563/clinexprheumatol/791fq8 36377563

